# Intestine specific regulation of pig cytidine-5′-monophospho-***N*-**acetylneuraminic acid hydroxylase gene for *N*-glycolylneuraminic acid biosynthesis

**DOI:** 10.1038/s41598-019-40522-9

**Published:** 2019-03-12

**Authors:** Kwon-Ho Song, Choong-Hwan Kwak, Tae-Wook Chung, Sun-Hyung Ha, Jun-Young Park, Ki-Tae Ha, Seung-Hak Cho, Young-Choon Lee, Cheorl-Ho Kim

**Affiliations:** 1 Molecular and Cellular Glycobiology Unit, Department of Biological Science, Sungkyunkwan University, Seoburo 2066, Jangan-Gu, Suwon, Gyunggi-Do 16419 Korea; 2 Division of Applied Medicine, School of Korean Medicine, Pusan National University, Yangsan City, Korea; 3 Korea National Institute of Health, Division of Bacterial Disease Research, 202, Osongsaengmyeong 2-ro, Osong-eup, Heungdeok-gu Korea; 4 Department of Medicinal Biotechnology, Dong-A University, Busan, 49315 Korea; 5 Samsung Advanced Institute for Health Sciences & Technology (SAIHST), Samsung Medical Center, Seoul, 06351 Korea

## Abstract

*N*-glycolylneuraminic acid (Neu5Gc), a generic form of sialic acid, is enzymatically synthesized by cytidine-5′-monophospho-*N*-acetylneuraminic acid hydroxylase (CMAH). Although expression of pig CMAH gene *pcmah* encoding CMAH has been reported to be regulated by pathogenic infection and developmental processes, little is known about the mechanisms underlying the regulation of *pcmah* gene expression. The objective of this study was to determine mechanism(s) involved in intestine specific regulation of *pcmah* gene by identifying several *cis*-acting elements and nuclear transcription factors that could directly interact with these *cis*-acting elements. We identified intestine specific promoter region (Pi) of *pcmah* gene located at upstream regions of the 5′flanking region of exon 1a and found that the promoter region is responsible for the transcriptional regulation of 5′*pcmah*-1. Based on reporter assays using serially constructed luciferase genes with each deleted promoter, we demonstrated that the Pi promoter activity was more active in intestinal IPI-2I cells than that in kidney PK15 cells, corresponding to both mRNA expression patterns in the two cell lines. In addition, we found that Sp1 transcription factor was necessary for basal activity of Pi promoter and that Ets-1 contributed to intestine-specific activity of Pi promoter. This study helps us understand transcriptional regulation of *pcmah* in the intestine of pig tissues. It also allows us to consider potential roles of Neu5Gc in interaction with environmental factors present in the intestinal tissue during pathogenic infection and developmental process.

## Introduction

Gastrointestinal tract lining functions as a barrier against hostile milieu such as foreign parasitic infection where epithelial cells are targets for infecting microbes and viruses^1^. Carbohydrates on the surface of intestinal epithelial cells have been implicated as the primary compounds that interact with microbes during the infection process. In particular, sialic acid (Sia) occupies the terminal position within glycan molecules and acts as the receptor for certain bacteria and viruses^2–5^.

The 9-carbon structure of Sia can be found in Deuterostome lineaged animals. It is frequently modified. One of modified structures of Sia is *N*-glycolylneuraminic acid (Neu5Gc) which is one of the most common Sia derivatives^6,7^. Neu5Gc is synthesized by cytidine-5′-monophospho (CMP)-N-acetylneuraminic acid hydroxylase (CMAH) which catalytically converts sugar nucleotide CMP-Neu5Ac to CMP-Neu5Gc^8–10^. Neu5Gc is a major xeno-antigen that is expressed in pigs and apes, but not in human due to genetic loss of CMAH in human^8–10^. However, it can be introduced into the human body by Neu5Gc-containing dietary sources, mainly red meat intake, leading to the production of anti-Neu5Gc antibodies in human serum^10–12^. It has been suggested that binding of Neu5Gc to anti-Neu5Gc antibodies is associated with the occurrence of chronic inflammatory diseases such as cancer and atherosclerosis^11–15^. Neu5Gc is considered as a tumor marker in various human cancers including colon carcinoma, retinoblastoma, breast cancer, and melanoma^15–17^. Neu5Gc also plays a vital role as a non-Gal antigen in immune antigenic rejection during pig-to-human xenotransplantation^18–20^. In addition, Neu5Gc acts as a target receptor for pathogens such as *Escherichia coli* K99 and for bacterial toxins such as subtilase cytotoxin secreted by Shiga toxigenic *E*. *coli*^3,21^.

Previous studies have shown that expression levels of Neu5Gc and CMAH gene are tissue-dependent^8,22^. In addition, the expression of CMAH gene is regulated by intracellular activation signals and disease status^23,24^. In mice, transcriptional mRNA expression of CMAH gene seems to be regulated by Gram-negative bacterial endotoxin and lipopolysaccharide (LPS)-induced mouse B-cell activation^25^. In the case of pigs, small intestinal synthesis of Neu5Gc in developing pigs is controlled by enzyme activity of hydroxylase and mRNA expression level of CMAH^24^. Although CMAH gene expression is basically regulated by development-derived factors and pathogenic infectious environments, the molecular mechanism(s) underlying the regulation of the CMAH gene, particularly in the viewpoint of directly control of Neu5Gc biosynthesis, is still unclear in any organism. In a recent report describing Sia levels in porcine milk during lactation, Neu5Ac has been found to be the major form of Sia (93–96%) while Neu5Gc (3–6%) is a minor form based on their contents irrespective of milk lactation stage^26^. However, Neu5Gc was unexpectedly the major Sia form in the porcine spleen (67–79%) and lung (36–49%) based on their contents. Skeletal muscle contained the lowest concentration of Neu5Gc among porcine tissues. In addition, all other organs have been found to contain higher levels of NeuGc than skeletal muscles during development^27^.

In a previous report^22^, our group has presented the complete pig CMAH gene *pcmah* with two 5′ alternative transcription variants (5′*pcmah*-1 and -2). Additionally, there are strong evidences for the existence of alternatively working promoters for *pcmah*^22^. Tissue specific expression pattern of *pcmah* suggests that its expression is regulated by alternative promoter utilization in a tissue-specific manner. Based on the dual existence of Neu5Ac and Neu5Gc in pig tissues, the biological role of alternative splicing of *pcmah* is likely to be important for their functions in endogenous and exogenous responses. Therefore, differences in Neu5Gc biosynthesis or CMAH enzyme activity in various pig tissues need to be clarified. Of the two alternative promoters of *pcmah*, promoter P2 has been demonstrated to be responsible for the expression of house-keeping gene. Hence, it was named promoter Ph^28^.

The objective of this study was to explain mechanism(s) involved in intestine specific regulation of *pcmah* gene through identification of *cis*-acting elements and nuclear transcription factors. In the present study, we identified an intestine specific promoter region and named it Pi promoter of the 5′flanking region of exon 1a. We further found that this intestine specific promoter was responsible for cell specific expression pattern of 5′*pcmah*-1. We finally demonstrated that Sp1 and Ets-1 transcription factors were necessary for basal activity and intestine specific activity of the intestine specific promoter of *pcmah*, respectively. This study helps us understand transcriptional regulation of *pcmah* in different pig tissues. It may contribute to our understanding of differential expression of Neu5Gc in different tissues of nonhuman animals.

## Results

### Identification of intestine specific promoter (Pi) of *pcmah*

**Figure 1:**
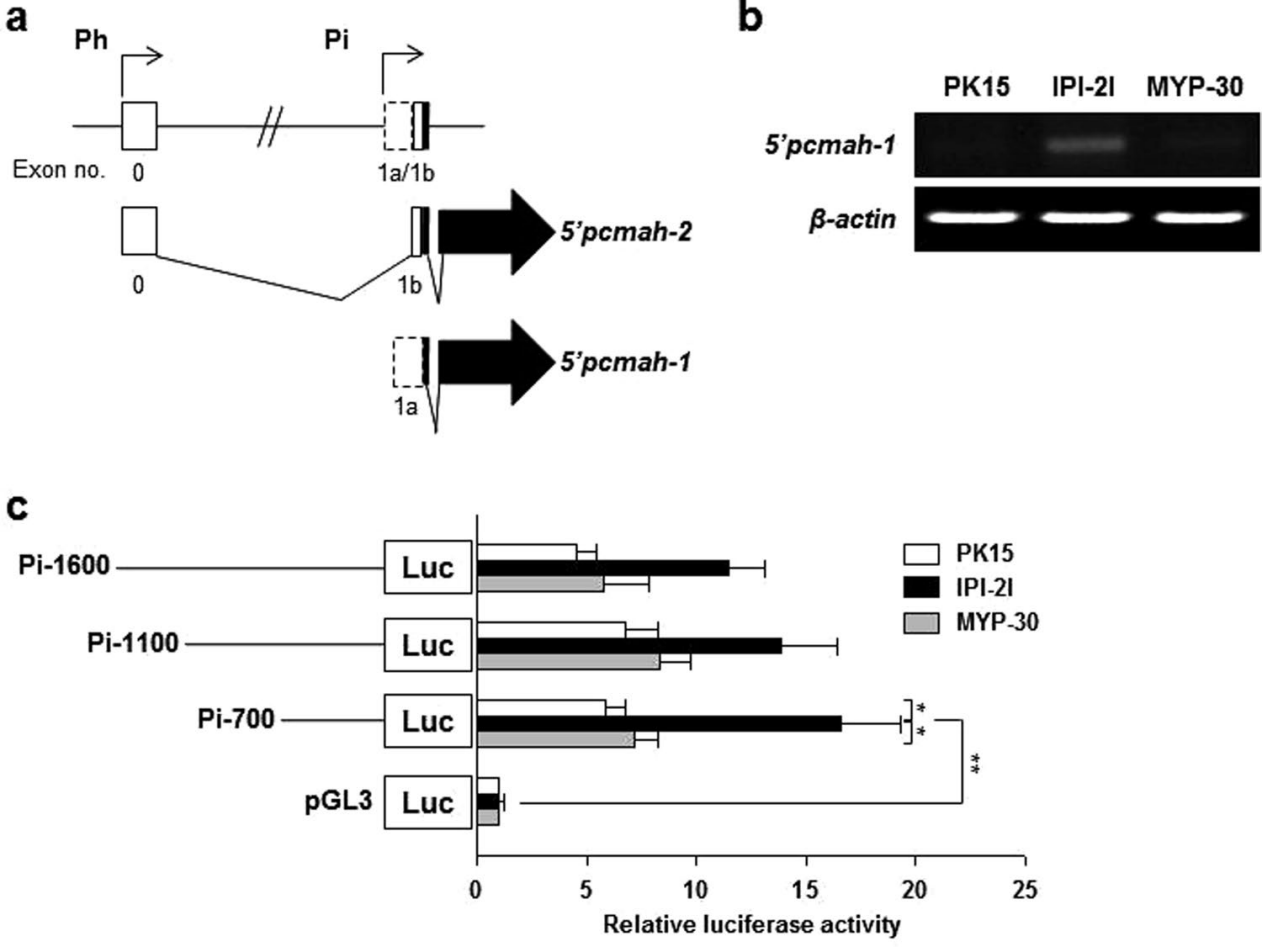
Identification of intestine specific promoter (Pi) of the *pcmah* gene. (**a**) Genomic structure of *pcmah*. Ubiquitous housekeeping promoter region of *pcmah* plays a role in the expression of 5′*pcmah*-2. Putative promoter region of the intestine specific splicing variant of *pcmah* (5′*pcmah*-1) is indicated by ‘Pi (intestine specific promoter)’. Shaded boxes indicate the coding exons, while open boxes indicate untranslated exons. Two splicing variants of *pcmah* share a common ORF region (shaded arrow boxes). (**b**) The relative levels of 5′*pcmah*-1 expression were measured and analyzed by RT-PCR in three different cells of PK15, IPI-2I, and MYP30. *β*-Actin was used as a positive control. (**c**) The relative reporter activities of the luciferase reporter plasmids with the proximal region of exon 1a in PK15, IPI-2I, and MYP30 cells. DNA fragments containing various lengths of the Pi promoter region, which is the proximal region of exon 1a, were subcloned into a PGL3-basic vector (pGL3), and each generated plasmid was transiently transfected into the three different pig cells of PK15, IPI-2I, and MYP30. Two enzyme activities of luciferase and *β*-Gal in each of the transfected cells were assayed as described in the ‘Materials and Methods’ section. In each transfected cell, the luciferase enzyme activity was normalized with the measured β-Gal activity. The relative fold value was then calculated from the ratio of the normalized activity and the activity in the empty pGL3-basic vector-transfected cells. Statistic bars represent the mean ± SE of three independent determinations. Differences in the fold value of luciferase enzyme activities were statistically analyzed using the Student *t*-test: *p < 0.05; **p < 0.01.

Results obtained from promoter analysis clearly showed that pig CMAH gene *pcmah* had two different splicing forms (5′*pcmah*-1 and -2) of promoters as 5′alternative forms (Fig. 1a). It exhibited a tissue-specific pattern of expression. The 5′*pcmah*-1 splicing form was mainly expressed in the intestinal tissue while the 5′*pcmah*-2 form was ubiquitously expressed in most tissues^28^. In order to investigate whether tissue specific expression of 5′*pcmah*-1 was relevant to cell type derived from different tissues or organs, we analyzed mRNA levels of 5′*pcmah-1* in pig kidney-derived PK15 cells, pig small intestine-derived IPI-2I cells, and pig aorta-derived MYP30 cells. 5′*pcmah*-1 was specifically expressed in IPI-2I cells, but not in PK15 or MYP30 cells (Fig. 1b). This result corresponds with a previous result that shows tissue specific expression pattern of 5′*pcmah-1*^28^, suggesting that 5′*pcmah*-1 is an intestine specific splicing variant. In order to investigate the molecular mechanisms involved in 5′*pcmah*-1 regulation, we isolated the 5′flanking region of exon 1a of *pcmah* and named it promoter Pi (intestine specific promoter) (Fig. 1a,c). To analyze Pi promoter, three fragments (1600, 1100, and 700) were recombinantly inserted into pGL3 basic vector and then transfected into PK15, IPI-2I, and MYP30 cells, respectively. The overall activity of Pi promoter in IPI-2I cells was higher than that in PK15 cells or MYP30 cells (Fig. 1c). These results also corresponded with cell-specific expression patterns of these two 5′*pcmah*-1 and -2 transcripts (Fig. 1b), suggesting that the Pi promoter could confer intestine-specific activity for expression regulation of 5′*pcmah*-1.

### Analysis of regulatory elements and characterization of Pi promoter

**Figure 2:**
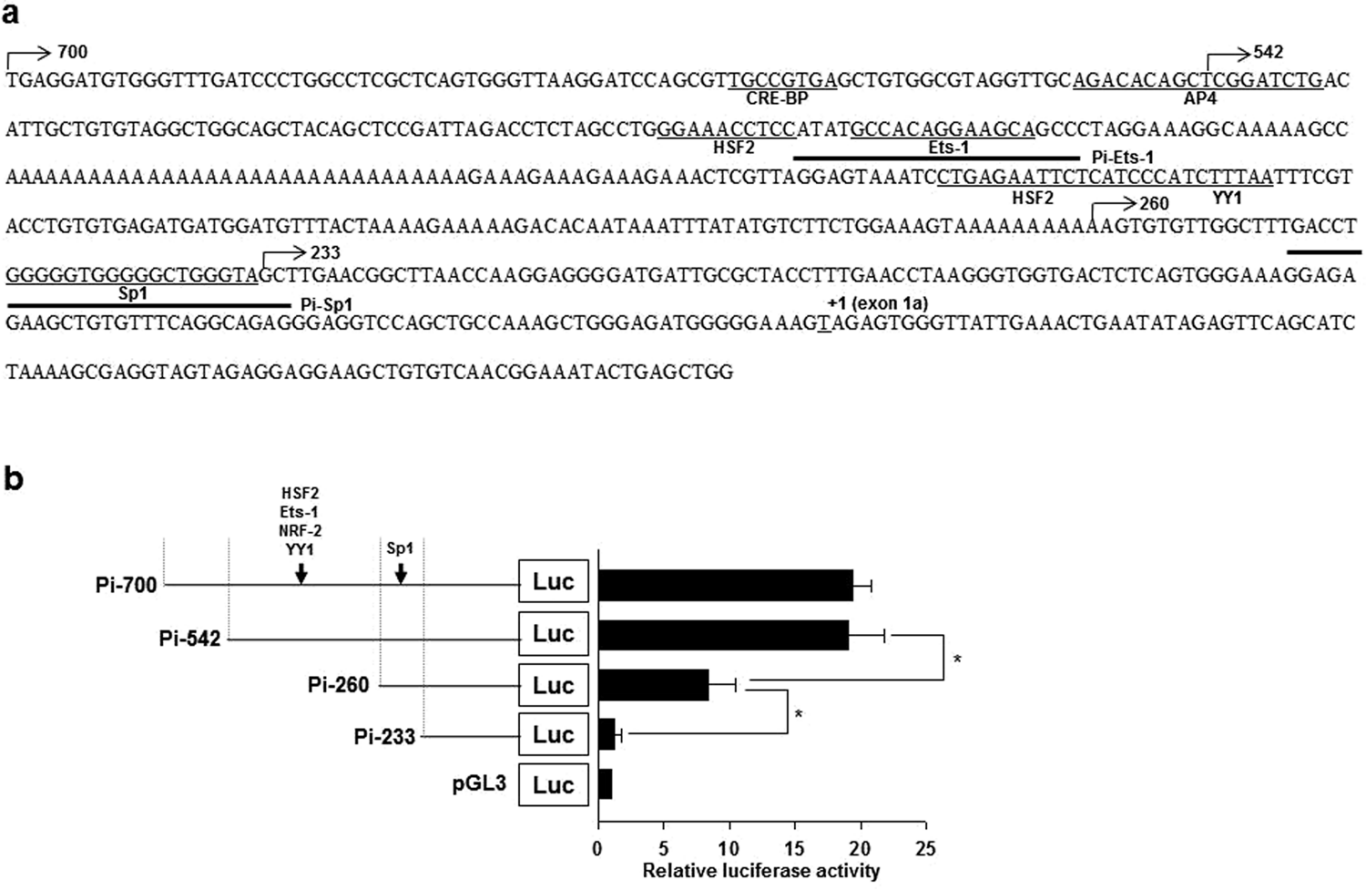
Detailed characterization and analysis of the Pi promoter region. (**a**) Analysis of nucleotide sequences of the Pi promoter region. For the transcription factors to interact, putative binding sites as DNA sequences are underlined. The end point of 5′deletion mutants is indicated by arrows. The position ‘+1’ indicates the transcription start site of 5′pcmah-1. Thick underlines indicate the oligonucleotide DNA sequences for the EMSA experiment. (**b**) The 5′deletion analysis of the Pi promoter region of pcmah in IPI-2I cells. Transcription factors, which exist in the region and are indicated by black arrows, are represented. Statistic bars represent the mean ± SE obtained from three independent determinations. Differences in the fold value of luciferase enzyme activities were statistically analyzed using the Student *t*-test: *p < 0.05.

Because Pi-700 fragment had the highest activity in IPI-2I cells, it was further analyzed for promoter characterization in IPI-2I cells. This promoter lacked TATA and CCAAT boxes. Instead, several polyA repeat sequences were present in Pi promoter (Fig. 2a). We then identified putative binding sites for CRE-BP, AP-4, HSF-2, Ets-1, NRF-2, YY1, and Sp1 in Pi promoter region of *pcmah* (Fig. 2a). In order to elucidate the functional role of putative transcription factor binding sites in Pi promoter activity, serially constructed 5′-deletion mutants Pi-542, Pi-260, and Pi-233, in addition to Pi-700, were generated and analyzed for their promoter activities in IPI-2I cells. The deletion of 282 and 27 nucleotides from Pi-542 and Pi-260, respectively, decreased Pi promoter activity, suggesting that these regions might contain positive regulatory elements required for Pi promoter activity (Fig. 2b). When these regions were assessed as transcription factor candidates, putative transcription factor elements including HSF2, Ets-1, NRF2, YY1, and Sp1 were found (Fig. 2b).

### Transcription factor Sp1 is crucial for basal activity of Pi promoter

**Figure 3:**
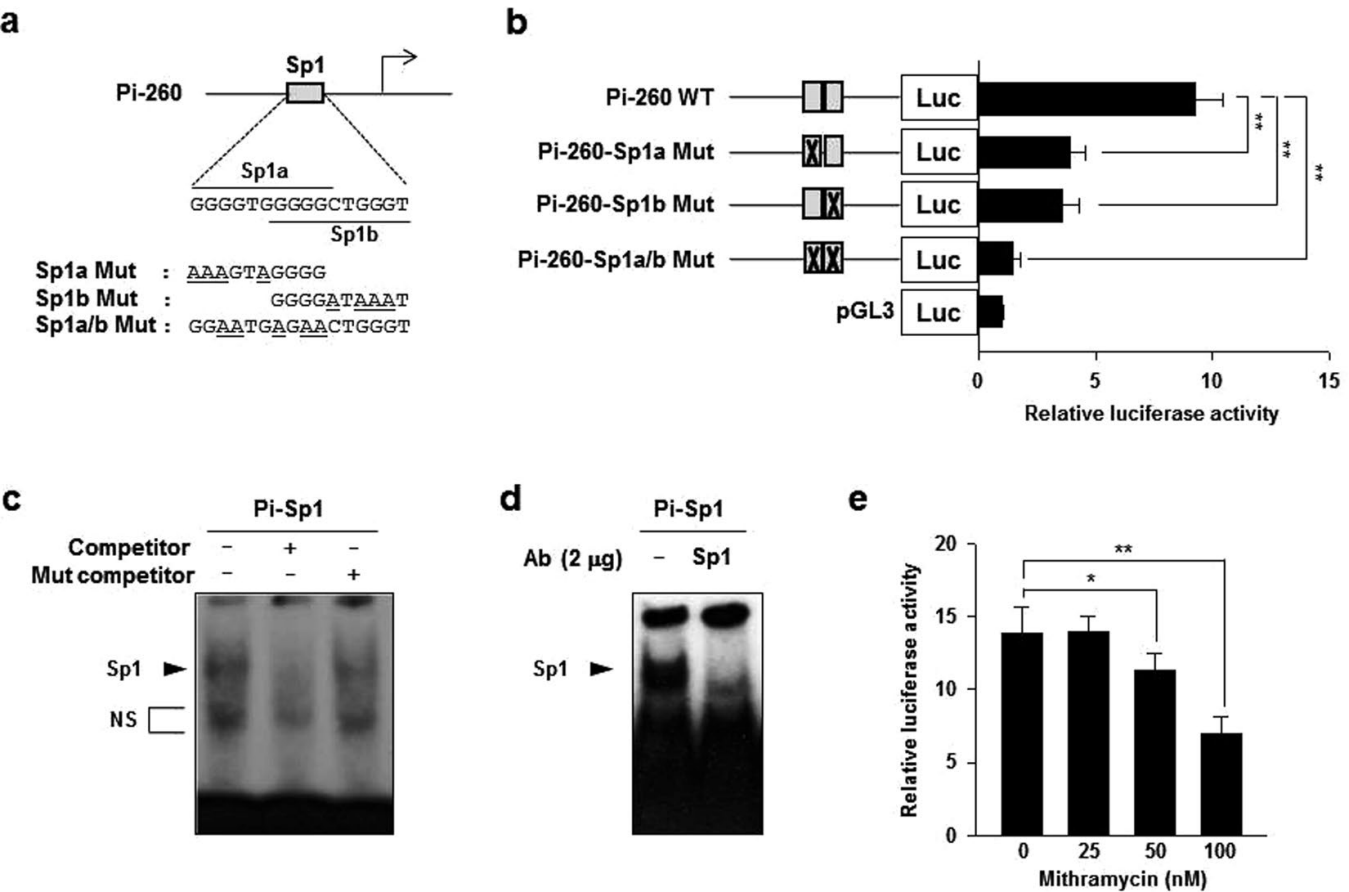
Transcription factor Sp1 is crucial for the basal activity of the Pi promoter. (**a**) Upper panel: nucleotide sequence of putative Sp1 binding sites (named here as Sp1a (upper lined) and Sp1b (under lined)) in Pi-175. Under panel: mutated bases in three Sp1 mutants are underlined. (**b**) Luciferase enzyme activities of three site-directed mutants and wild type Pi-175 in IPI-2I cells. Gray boxes and crossed gray boxes indicate the wild type or mutation of the putative Sp1 binding site(s), respectively. (**c**,**d**) Nuclear extracts of IPI-2I cells were incubated with ^32^P labeled oligonucleotide DNA probes (Pi-Sp1) containing two overlapping Sp1 binding sites on the Pi promoter. The reaction mixture was then examined by EMSA as described in the ‘Materials and Methods’ section. (**c**) Competition binding assay was performed using a non-labeled probe DNA (competitor) or a non-labeled mutant DNA probe (Mut competitor) for the Sp1 binding site of the Pi promoter. (**d**) Super-shift assay using anti-Sp1 antibodies was then performed. The arrows indicate the specific binding complex for Sp1. ‘NS’ refers to non-specific bands. (**e**) The effect of Sp1 inhibitor on the P1 promoter activity. P1-175 plasmid was transiently transfected into IPI-2I cells and various concentrations (25, 50, and 100 nM) of mithramycin A were added to the cells for 20 h. Bars represent the mean ± SE obtained from three independent determinations. Differences in the fold value of luciferase enzyme activities were statistically analyzed using the Student *t*-test: *p < 0.05; **p < 0.01.

Previously, we have demonstrated that transcription factor Sp1 can specifically bind to its binding sites on housekeeping promoter (Ph) regions of *pcmah*. In addition, it positively regulates *pcmah* promoter activity in PK15 cells^28^. Interestingly, putative Sp1 binding sites overlapped by two Sp1 binding sites in Pi-260 fragment (Fig. 3a). We questioned whether Sp1 binding sites might be responsible for the basal activity of Pi promoter. In order to address this possibility, these putative Sp1 binding sites were experimentally altered by site-directed mutagenesis. Using altered clones, the effect of each mutation on promoter activity was examined in IPI-2I cells. In Pi promoter, luciferase activities of Pi-260 Sp1a Mut, Pi-260 Sp1b Mut, and Pi-260 Sp1a/b Mut (mutated in both Sp1a and Sp1b sites) were reduced by approximately 58% (9.2 to 3.9 folds), 62% (9.2 to 3.5 folds), and 84% (9 to 1.5 folds), respectively, compared to luciferase activity of wild type Pi-260 plasmid (Fig. 3b). Next, in order to confirm whether Sp1 could bind to Pi promoter *in vitro*, gel mobility shift assay was performed. Nuclear protein from IPI-2I cells was able to be bind to ^32^-P labeled oligonucleotide probe. Pi-Sp1 contained two overlapping putative Sp1 binding sites of the Pi promoter region (Fig. 3c). The specificity of binding was confirmed under experimental condition of competition assay which used 100-fold molar excess of each unlabeled oligonucleotide probe and each unlabeled mutant probe that carried point mutation in the Sp1 consensus sequence of the region. As expected, results clearly showed that the unlabeled probe efficiently competed for the binding of the transcription factor to labeled oligonucleotide probe. In contrast, unlabeled mutant probe could not compete for the binding of the transcription factor (Fig. 3c). To further clarify whether the bound complex could be linked to transcription factor Sp1 protein, a super-shift assay was performed in the presence of anti-Sp1 antibody. Super-shift band was not detected in the assay. However, the binding intensity of the major Sp1 complexed form was dramatically decreased by treatment with anti-Sp1 specific antibody, indicating competitively specific binding by Sp1 (Fig. 3d). To demonstrate that transcription factor Sp1 could directly regulate Pi promoter activity of the gene, effects of mithramycin A, an Sp1 inhibitor, on Pi promoter was examined. As expected, results showed that treatment with increasing concentrations (25 nM to 100 nM) of mithramycin A decreased luciferase activity of Pi-260 in a dose-dependent manner (Fig. 3e). These results clearly suggest that Sp1 can bind to its putative *cis*-binding sites on the Pi promoter region of *pcmah* and that it is crucial for basic transcriptional regulation of Pi promoter activity.

### Ets-1 is essential for intestine specific activity of Pi promoter

**Figure 4:**
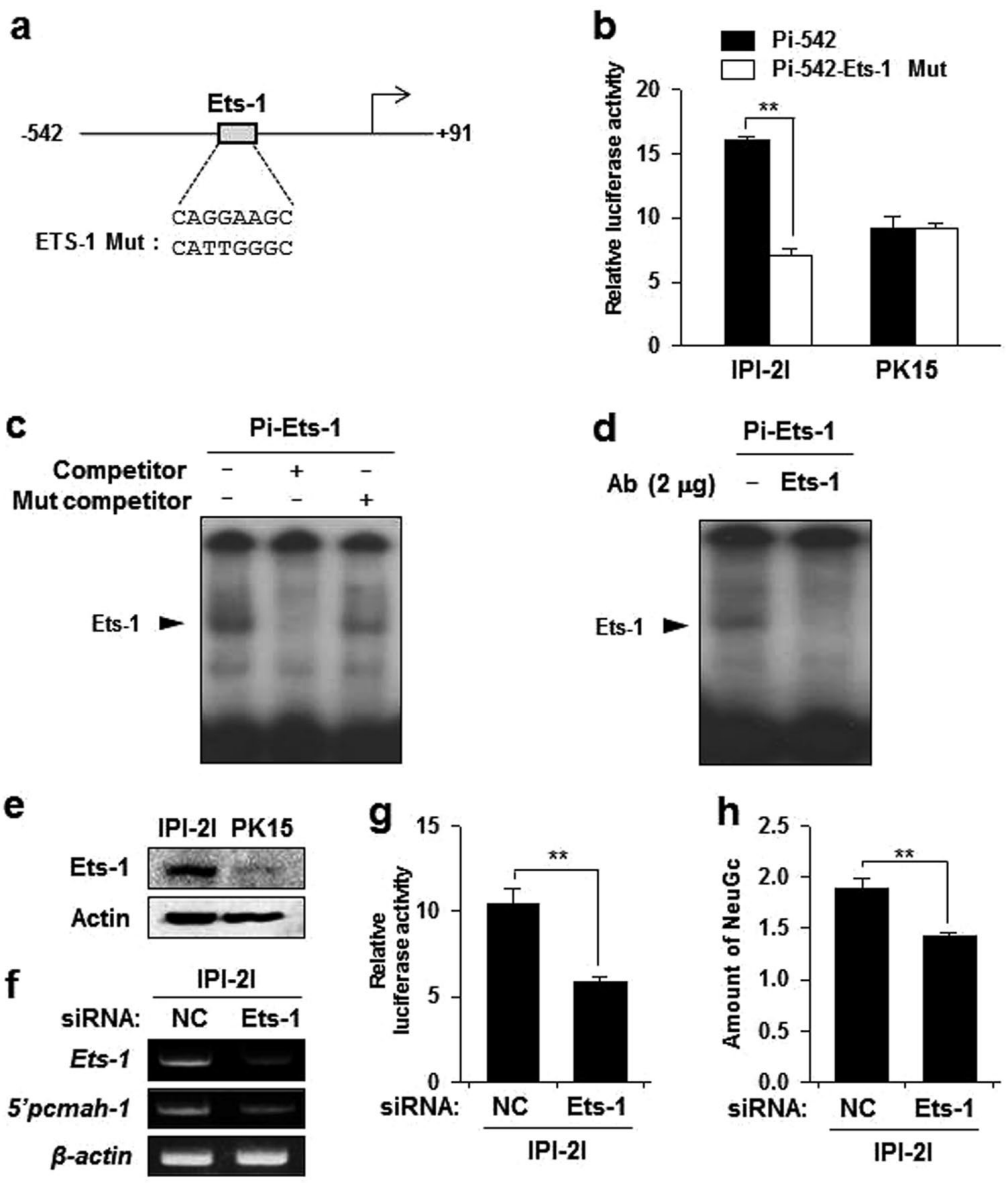
Ets-1 is essential for intestine specific activity of the Pi promoter. (**a**) Upper panel: nucleotide sequence of the putative Ets-1 binding site in Pi-457. Under panel; mutated bases in the Ets-1 mutant are underlined. (**b**) Luciferase activities of Ets-1 mutant and wild type of Pi-457 in IPI-2I and PK15 cells. (**c**,**d**) Nuclear extracts of IPI-2I cells were incubated with ^32^P labeled oligonucleotide probes (Pi-Ets-1). The mixture was subsequently examined by EMSA as described in the ‘Materials and Methods’ section. (**c**) Competition binding assay was performed using a non-labeled probe (competitor) or a non-labeled mutant probe (Mut competitor) for Ets-1 binding site of the Pi promoter. (**d**) Super-shift assay using anti-Ets-1 antibodies was performed. The arrows indicate the specific binding complex for Ets-1. (**e**) Expression level of Ets-1 in IPI-2I and PK-15 cells was analyzed by immunoblotting assay. Actin was included as an internal loading control. (**f**) IPI-2I cells were transfected with negative control (NC) and siRNAs targeting Ets-1. The gene expression levels of Ets-1 and 5′pcmah-1 were analyzed by RT-PCR in IPI-2I cells. (**g**) Luciferase activities of Pi-457 in the siRNA transfected IPI-2I and PK15 cells. Bars represent the mean ± SE obtained from three independent determinations. (**h**) The level of NeuGc in Ets-1 siRNA-transfected IPI-2I cells was measured by ELISA assay. Differences in the fold value of optical density were statistically analyzed using the Student *t*-test: **p < 0.01.

In addition to Sp1, genetic deletion of the region containing putative binding sites for transcription factor HSF2, Ets-1, NRF2, and YY1 markedly decreased the luciferase activity of Pi promoter (Fig. 2b). Among them, Ets-1 transcription factors are widely expressed in developing and mature intestine^29^. For further characterization of possible intestine-specific *cis*-elements in Pi promoter, we investigated the role of putative Ets-1 binding sites in Pi promoter. Interestingly, mutation of Ets-1 binding site on Pi-542 significantly decreased the activity of Pi-542 in intestine IPI-2I cells, but not in kidney PK15 cells (Fig. 4a,b), suggesting that the putative Ets-1 binding site might act as a *cis*-acting element to confer intestine specific activity of the Pi promoter. To determine whether Ets-1 could directly interact with putative Ets-1 binding sites in the Pi promoter, electrophoretic mobility shift assay (EMSA) was performed. Nuclear protein isolated from IPI-2I cells was able to bind to ^32^-P labeled Pi-Ets-1oligonucleotide probe (Fig. 4c). In addition, unlabeled probe was efficiently competitive for binding of Ets-1 to the labeled Pi-Ets-1 probe while no competition was observed for unlabeled mutant probe (Fig. 4c). A super-shift assay further verified the specificity of Ets-1 and DNA interaction (Fig. 4d). These results indicate that Ets-1 can specifically bind to its putative binding sites on the Pi promoter region of *pcmah*.

We reasoned that the dependency difference in Ets-1 binding site on IPI-2I and PK15 cells might be due to the spontaneous expression level of Ets-1. Thus, we compared protein levels of Ets-1 in IPI-2I and PK-15 cells. Indeed, Ets-1 protein level in IPI-2I cells was higher than that in PK15 cells (Fig. 4e). This suggests that Ets-1 is a *trans*-acting element that confers intestine-specificity of *pcmah* to IPI-2I cells. To further investigate the role of EtS-1 in *pcmah* expression, we transfected siRNAs targeting Ets-1 into IPI-2I cells. Notably, knockdown of Ets-1 decreased 5′*pcmah*-1 expression as well as luciferase activity of Pi-542 in IPI-2I cells (Fig. 4f,g). Furthermore, the amount of NeuGc was decreased in Ets-1 siRNA transfected IPI-2I cells as compared to that in NC siRNA-transfected cells (Fig. 4h). These results suggest that Ets-1 is essential for intestine specific expression of *pcmah*.

## Discussion

During the course of evolution from their ancestors, mice and pigs shared a last common ancestor over 98 million years ago, with a large difference in the expression of a specific sialic acid called Neu5Gc. Neu5GC is widely expressed on most mammalian tissues, but not on human cells^30^. Apart from humans, recent studies have shown that several other groups of mammals have also lost the activity of CMAH. Mustelids (ferrets and relatives) and pinnipeds (seals, sea lions and walruses) lack an active CMAH. Consequently, they do not produce Neu5Gc^31^, although they belong to highly social species with very high chance of microbial infections. Ferret CMAH gene has been suggested to be deleted by an ancient mutation that is also shared by other members of the order Carnivora^31^. The exclusive expression of Neu5Ac in ferrets may contribute to their suitability as a model for human-adapted infectious diseases. Similarly, a CMAH mutation has also been known to occur in new world monkeys, differentiating them from old world monkey species which express Neu5Gc^32^. Compared to macaques (an old-world monkey species), marmosets (a new-world monkey species) have also been shown to be suitable for human-adapted transmission studies because of their small sizes requiring low doses for potential vaccine and drug studies and expression of symptoms similar to those seen in influenza virus-infected humans^33^.

Previously, many reports have demonstrated that expression levels of Neu5Gc and CMAH are regulated during infection by intestinal parasites and during developmental process^2–5,34^. In rats, Neu5Gc level is down-regulated by a decrease in CMAH mRNA expression during infection with *Nippostrongylus brasiliensis*^1^. In pigs, ganglioside GM3 (Neu5Gc) or Neu5Gc content is maximal at birth and gradually decreased in adults. This may explain the susceptibility of newborn piglets to pig enteric pathogens such as *E*. *coli* K99^4,21,35,36^. These observations suggest that Neu5Gc production can be regulated at transcriptional level during infection and developmental processes. However, little is known about the mechanisms underlying the regulation of CMAH gene. Since intestine tissues are important for selective expression of this gene in response to pathogenic infection in pigs, intestine specific regulation of the promoter is in the best interest of pig Neu5Gc biosynthesis. In the present study, we elucidated the mechanism(s) involved in intestine specific regulation of the pig CMAH gene by identifying *cis*-acting elements and their interacting transcription factors crucial for *pcmah* intestinal promoter activity. Such mechanisms for *pcmah* transcriptional regulation may give us insight into the mechanism(s) of Neu5Gc regulation during infectious and developmental processes.

In our previous studies^22,28^, we have demonstrated that *pcmah* has two distinct 5′alternative splicing forms, namely 5′*pcmah*-1 and -2. We have also shown that *pcmah* expression has a tissue specific pattern such that 5′*pcmah*-1 is mainly expressed in the intestine while 5′*pcmah*-2 is rather ubiquitously expressed in most tissues^22,28^. The two alternative mRNA forms specifically expressed in the tissues are strictly corresponded with those observed in the PK15 and IPI-2I cells, which were derived from pig kidney tissue and small intestine tissue, respectively^22,28^. Tissue specific gene expression characterized by transcripts incorporating alternative 5′ ends is known to be under the regulation of multiple promoters^37–39^. In this study, we isolated the intestine-specific promoter (Pi) of *pcmah*. Based on luciferase reporter assay, we demonstrated that the Pi promoter, relative to the upstream region of 5′*pcmah*-1, was mainly active in IPI-2I cells (Fig. 2). These results corresponded with mRNA expression patterns of 5′*pcmah*-1 shown in Fig. 1b, suggesting that these alternative promoters might confer differences in the regulation of *pcmah* transcription in pig tissues. However, PK15 cells have only basal activity of the Pi promoter and expression of *pcmah*-1 mRNA by promoter Pi was scarcely detected. Here, we used IPI-2I cells to further investigate elements related to Pi promoter activity. Based on the present results, transcription factor Sp1 can specifically bind to its putative binding sites on Pi promoter regions of *pcmah* and positively upregulate promoter activity in cells. Although it is clear that Sp1 is essential for the basal activity of Pi promoter, more complex regulatory element(s) are required for its full activity.

In fact, serial 5′deletion analysis of Pi promoter region suggested that Ets-1 might additionally act as a positive regulatory element with Sp1 as evidenced by the fact that genetic deletion of DNA regions containing putative Ets-1 binding sites remarkably reduced reporter luciferase activity of Pi promoter. Mutation of Ets-1 *cis*-element or knockdown of the Ets-1 trans-element significantly disrupted P1 promoter activity in IPI-2I cells, but not in PK15 cells (Fig. 4b,g). Interestingly, it has been reported that Ets transcription factors are widely expressed in developing and mature intestines losely related to a certain inflammatory disease^29,40,41^. These results demonstrate that Ets-1 plays an important role in the activity of Pi promoter. Thus, it may contribute to the intestine-specific expression of *pcmah*.

**Figure 5:**
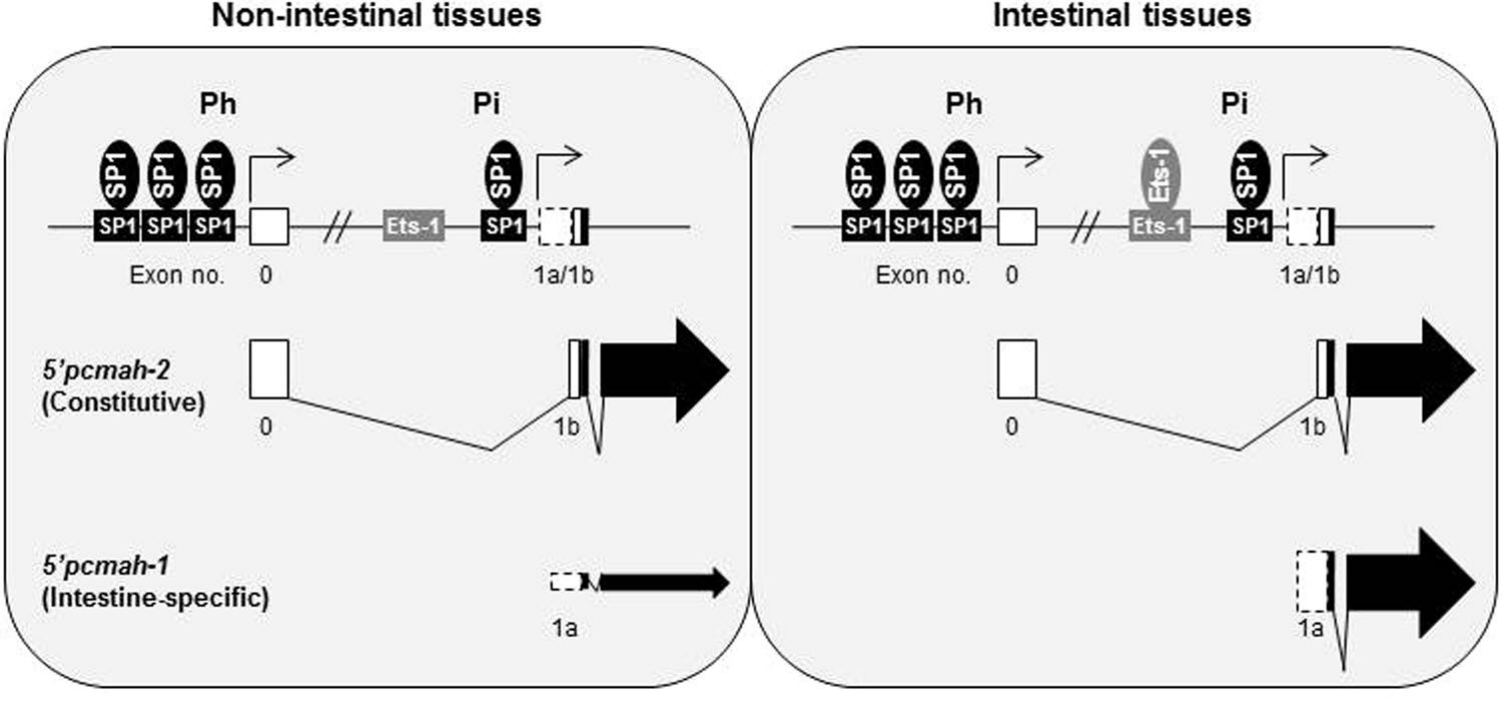
Schematic illustration of the intestine specific promoter (Pi) 5′*pcmah-1* and the house-keeping (constitutive) promoter (Ph) *5*′*pcmah*-2 of the *pcmah* gene. The housekeeping promoter region of *pcmah*, starting at the 5′ flanking region including 3 independent SP1 of Exon 0, regulates the constitutive expression of 5′*pcmah*-2. Intestine specific splicing variant of *pcmah* (5′*pcmah*-1) is stringently controlled by the intestine specific promoter P1, starting at the 5′ flanking region, including Ets-1 and SP1 of Exon 1a.

In conclusion, as schematically illustrated in Fig. 5, two 5′alternative splicing forms of pig CMAH gene, 5′*pcmah*-1 and -2, are controlled by Pi (intestine specific) and Ph (house-keeping) promoters, respectively. Furthermore, each splicing variant is stringently regulated by different transcriptional *cis*- and *trans*-acting elements on each promoter region. Sp1 transcription factor binds to its specific binding sites on Pi and Ph promoter regions and positively upregulates the promoter activity in pig cells derived from tissues. On the other hand, Ets-1 transcription factor which is abundant in intestinal tissues is important for the activity of Pi promoter and expression of 5′*pcmah*-1, indicating that it may contribute to intestine-specific expression of *pcmah*. These findings provide enhanced insight into molecular regulation of CMAH gene expression at transcriptional level as well as new insights into the regulatory mechanisms of Neu5Gc biosynthesis during pathogenic infectious events and embryonic developmental stages.

## Materials and Methods

### Cell culture

Dulbecco′s modified Eagle’s medium (DMEM; WelGENE, Daegu, Korea) was used for culturing PK15 pig kidney cells. IPI-2I cells derived from pig small intestinal tissue were cultured in DMEM containing 0.024 IU/ml insulin and 4 mM glutamine as additives. MYP30 pig endothelial cells were cultured in DMEM containing 0.1 mMol/L nonessential amino acids. All culture media were supplemented with 10% heat-inactivated fetal bovine serum (FBS; WelGENE, Korea), 100 unit/ml penicillin, and 100 μg/ml streptomycin. Cell cultures were routinely maintained at 37 °C with 5% CO_2_ atmosphere.

### RT-PCR

Total RNAs were isolated using commercially available TRIZOL reagent (Invitrogen, USA) and cDNA synthesis was performed using AccuPower^®^ RT-PreMix kit (Bioneer, Daejon, Korea) according to the manufacturer’s protocol. PCR was performed with EF-Taq polymerase (SolGent, Korea) using the following specific primers: 5′*pcmah*-1, 5′-GTCAACGGAAATACTGAGCTGGGT-3′ (forward) and 5′-TCGTCTTGACAGAAGCTTCCAGGA-3′ (reverse); Ets-1, 5′-AGAGTCCCGCTATACCTCGGA-3′ (forward) and 5′-AGGCTTGTCCTTGTTGAGGTC-3′ (reverse); β-*actin*, 5′-CACGCCATCCTGCG TCTGGA-3′ (forward) and 5′-TCTGCATCCTGTCGGCGATG-3′ (reverse). For the generalization of obtained data, equal amounts of mRNA were used. Expression levels of β-*actin* as internal control were analyzed and confirmed.

### DNA constructs

Fragments of different lengths in the 5′-flanking region of *pcmah* were amplified by PCR using pig BAC clone library which contained *pcmah* genome or cloned 5′-flanking DNAs as template with primer sets. Primer sequences are listed in Table 1. Obtained PCR products were digested using *Bgl* II and *Hind* III. Digested PCR products were then ligated with *Bgl* II and *Hind* III restriction sites of pGL3-Basic Vector as a vehicle (Promega, USA).

**Table 1:** DNA sequences of primers and probes used in this study.

*Primers for* 5′ *Deletion mutants*^*a*^	
Pi-1600(Bgl II-F)	ACAGATCTTGAAGGACAGCTTTGGCTCT
Pi-1100(Bgl II-F)	ACAGATCTAAGGATAACCTGACCCCTTTGC
Pi-700(Bgl II-F)	ACAGATCTTGAGGATGTGGGTTTGATCC
Pi-542(Bgl II-F)	ACAGATCTTCGGATCTGACATTGCTG
Pi-260(Bgl II-F)	ACAGATCTAAGTGTGTTGGCTTTGACCTG
Pi-223(Bgl II-F)	ACAGATCTAGCTTGAACGGCTTAACCAAG
Pi(Hind III-R)	ACAAGCTTCAGTATTTCCGTTGACACAG
**Primers for Direct mutagenesis** ^**b**^	
Pi-SP1a MutF	TTGACCTGaaaGTaGGGGCTGGGTAGCTTGAAC
Pi-SP1a MutR	TACCCAGCCCCtACtttCAGGTCAAAGCCAACA
Pi-SP1b MutF	GGTGGGGGaTaaaTAGCTTGAACGGCTTAACC
Pi-SP1b MutR	TCAAGCTAtttAtCCCCCACCCCCAGGTCAAAG
Pi-SP1ab MutF	TTGACCTGGGaaTGaGaaCTGGGTAGCTTGAACG
Pi-SP1ab MutR	CTACCCAGttCtCAttCCCAGGTCAAAGCCAAC
Pi-Ets-1 MutF	TATGCCACAttggGCAGCCCT
Pi-Ets-1 MutR	AGGGCTGCccaaTGTGGCATA
**Probes for EMSA** ^**b**^	
Pi-SP1	ACCTGGGGGTGGGGGCTGGGTAGCTT
Pi-SP1 Mut	ACCTGGGGGTGGGGGaTaaaTAGCTT
Pi-Ets-1	TATGCCACAGGAAGCAGCCCT
Pi-Ets-1 Mut	TATGCCACAttggGCAGCCCT

^a^Restriction enzyme cleavage sites were underlined.

^b^Lowercase letters indicate mutated nucleotides sequences.

### siRNA constructs

Synthetic small interfering RNA (siRNA) specific for Ets-1 were purchased from Genolution (Korea): Negative control (NC), 5′-CCUCGUGCCGUUCCAUCAGGUAGUU-3′ (sense), and 5′-CUACCUGAUGGAACGGCACGAGGUU-3′ (antisense); Ets-1, 5′-GGAAUUACUCACUGA UAAAUU-3′ (sense) and 5′-UUUAUCAGUGAGUAAUUCCUU-3′ (antisense). siRNA was delivered into 6-well plates at a dose of 100 pmol per well using commercially available WelFect-EXTM PLUS Transfection Reagent (WelGENE, Daegu, Korea).

### Site directed mutagenesis

Site directed DNA mutagenesis was performed using a QuickChange XL Site-directed Mutagenesis kit (Agilent, USA) according to the manufacturer’s instructions. Primer sequences are listed in Table 1. These primers were used to introduce desired DNA mutations to constructs of *pcmah* promoter. Multiple transcription factor binding sites were subjected to mutation from single mutation constructs by repeated mutagenesis with a different primer set. These generated mutation constructs were confirmed by DNA sequencing analysis.

### Luciferase assay

Cells were cultured after they were seeded onto 12-well culture plates one day prior to luciferase assay. Each cell was co-transfected with each luciferase reporter construct and β-galactosidase reporter plasmid using WelFect-EXTM PLUS Transfection Reagent (WelGENE, Korea). After 24 h of culturing, cells were harvested and subjected to cell lysis with 1 X Passive lysis buffer (Promega, USA). Enzyme activities of Luciferase and β-galactosidase were measured using luciferase and *β*-galactosidase activity assay system (Promega, USA). Luciferase enzyme activity was then normalized against β-galactosidase enzyme activity in cell lysate and calculated as an average of three sets of independently performed experiments.

### EMSA

For EMSA, single-stranded oligonucleotides were commercially synthesized by IDT DNA (IDT, USA). DNA sequences of these synthesized probes are listed in Table 1. To generate double stranded DNA probes, complementary oligonucleotides were mixed together at a 1:1 molar ratio and subjected to heating at 95 °C for 2 min followed by cooling down to 25 °C for 45 min. To obtain nuclear protein of each cell, each nuclear extract was prepared and isolated as previously described^42^. EMSA experiment was performed using a gel shift assay system kit (Promega, USA) according to the supplier’s recommended protocol and instructions. Briefly, 1.75 pmol/μl of double-stranded oligonucleotide probe was end-labeled with [γ-^32^P]ATP (3000 Ci/mmol; BMS, USA) using T4 polynucleotide kinase (Promega, USA). For protein-DNA binding, reaction mixture contained 2 μg of nuclear extract and 2 μl of gel shift binding buffer [4% glycerol, 1 mM MgCl_2_, 0.5 mM EDTA, 0.5 mM DTT, 50 mM NaCl, 10 mM Tris-HCl (pH 7.5), and 0.05 mg/ml poly (deoxyinosine-deoxycytosine)] with or without unlabeled wild type (wt) or mutant double-stranded oligonucleotide for binding competition. After preincubating at room temperature for 10 min, the reaction mixture was further incubated with labeled probe for 20 min at room temperature. For antibody competition assay, the nuclear extract was incubated with 2 μg of anti-Sp1 (Santa Cruz, USA) or Ets-1 antibody (Santa Cruz, USA) for 8 h at room temperature or overnight at 4 °C in the presence of binding buffer prior to addition of the labeled probe. The incubated reaction mixture was then separated by electrophoresis gel using 4% nondenaturing polyacrylamide in 0.5 × Tris-borate EDTA buffer at 250 V for 30 min. The separated polyacrylamide gel was dried for detection by autoradiography.

### Immunoblot Analysis

IPI-2I and PK-15 cells were rinsed twice with PBS and lysed with RIPA lysis buffer containing 20 mM Tris/HCl pH 7.5, 150 mM NaCl, 1% Triton X-100, 2 mM EDTA, 10% glycerol, 0.1% SDS, and 0.5% sodium deoxycholate with protease inhibitor cocktail (1 mM Na_3_VO_4_, 20 μg/mL PMSF, 10 μg/μL leupeptin, and 50 mM NaF). Lysates were centrifuged at 13,000 rpm for 10 min. Concentrations of whole cell lysates were measured using Lowry assay (Bio-Rad, Hercules, CA, USA). Then 25 μg of total proteins were separated by sodium dodecyl sulfate-polyacrylamide gel electrophoresis (SDS-PAGE) and transferred onto nitrocellulose membranes using a Hoefer electrotransfer system (Amersham Biosciences, Amersham, UK). These transferred nitrocellulose membranes were incubated with anti-Ets1 and anti-β-actin antibodies (1:1000 dilution in 1% skim milk) at 4 °C overnight followed by incubation with a secondary antibody (1:5000 dilution in 1% skim milk) for 1 h at room temperature. Bands were visualized by enhanced chemiluminescence (Sigma-Aldrich) and analyzed using ChemiDoc (Davinchi-K, Seoul, Korea). Polyclonal rabbit anti Ets-1 and polyclonal mouse anti-actin antibodies were purchased from Santa Cruz Biotechnology (Santa Cruz, CA, USA). Secondary antibodies were horseradish peroxidase conjugated anti-mouse IgG and anti-rabbit IgG (Thermo Scientific Pierce, Rockford, IL, USA).

### Enzyme linked immunosorbent assay (ELISA)

ELISA was performed using an Anti-Neu5Gc antibody Kit (BioLegend, San Diego, CA, USA), according to the supplier’s recommended instruction. Whole cell lysates were coated onto ELISA plates overnight at 4 °C. Wells were then washed with PBS. After blocking, the plate was incubated with chicken polyclonal IgY anti-Neu5Gc antibody (1:1000) for three hours at ambient temperature. The plate was then incubated with *Horseradish* peroxidase conjugated anti-chicken antibody (1:5000) for 1 h at ambient temperature. Developing reagent, 3,3′,5,5′-tetramethylbenzidine, was then added to the plate and optical density value was measured at 450 nm.

## Supplementary information

English Editing Certificate
