# Supplementary material for: Intestine specific regulation of pig cytidine-5′-monophospho-N-acetylneuraminic acid hydroxylase gene for N-glycolylneuraminic acid biosynthesis
Source: Sci Rep. 2019 Mar 12;9:4292. doi: 10.1038/s41598-019-40522-9 (PMC6414617; doi:10.1038/s41598-019-40522-9)
Supplement: Supplementary file 1 — English Editing Certificate [file 41598_2019_40522_MOESM1_ESM.pdf]

CERTIFICATE OF EDITING

HARRISCO
TOP EDITING COMPANY FOR
ACADEMIC RESEARCH PAPERS

Scientific English Research Paper Editing Service

1108, Hwanghwa B/D, 832-7, Yeoksam-Dong Kangnam-Ku

Seoul, the Republic of Korea

Tel : 82-2-557-1810~1

http://en.harrisco.net

The following manuscript was proofread and edited by
the professional English editors at HARRISCO.

Manuscript Title :

Intestine specific regulation of pig cytidine-5â€™-monophospho-N-acetylneuraminic acid hydroxylase gene for N-glycolylneuraminic acid biosynthesis

Manuscript Authors :

KIM, CHEORL-HO

Date of Issue: :

February 01, 2019

Yours truly,

HARRISCO
